# Validity of a minimally invasive autopsy for cause of death determination in maternal deaths in Mozambique: An observational study

**DOI:** 10.1371/journal.pmed.1002431

**Published:** 2017-11-08

**Authors:** Paola Castillo, Juan Carlos Hurtado, Miguel J. Martínez, Dercio Jordao, Lucilia Lovane, Mamudo R. Ismail, Carla Carrilho, Cesaltina Lorenzoni, Fabiola Fernandes, Sibone Mocumbi, Zara Onila Jaze, Flora Mabota, Anelsio Cossa, Inacio Mandomando, Pau Cisteró, Alfredo Mayor, Mireia Navarro, Isaac Casas, Jordi Vila, Maria Maixenchs, Khátia Munguambe, Ariadna Sanz, Llorenç Quintó, Eusebio Macete, Pedro Alonso, Quique Bassat, Jaume Ordi, Clara Menéndez

**Affiliations:** 1 ISGlobal, Barcelona Centre for International Health Research (CRESIB), Hospital Clinic of Barcelona, Universitat de Barcelona, Barcelona, Spain; 2 Department of Pathology, Hospital Clinic of Barcelona, Universitat de Barcelona, Barcelona, Spain; 3 Department of Microbiology, Hospital Clinic of Barcelona, Universitat de Barcelona, Spain; 4 Department of Pathology, Maputo Central Hospital, Maputo, Mozambique; 5 Faculty of Medicine, Eduardo Mondlane University, Maputo, Mozambique; 6 Department of Gynecology and Obstetrics, Maputo Central Hospital, Maputo, Mozambique; 7 Centro de Investigação em Saúde de Manhiça, Maputo, Mozambique; 8 ICREA, Catalan Institution for Research and Advanced Studies, Pg. Lluís Companys, Barcelona, Spain; 9 Consorcio de Investigación Biomédica en Red de Epidemiología y Salud Pública (CIBERESP), Madrid, Spain; Umeå Centre for Global Health Research, Umeå University, SWEDEN

## Abstract

**Background:**

Despite global health efforts to reduce maternal mortality, rates continue to be unacceptably high in large parts of the world. Feasible, acceptable, and accurate postmortem sampling methods could provide the necessary evidence to improve the understanding of the real causes of maternal mortality, guiding the design of interventions to reduce this burden.

**Methods and findings:**

The validity of a minimally invasive autopsy (MIA) method in determining the cause of death was assessed in an observational study in 57 maternal deaths by comparing the results of the MIA with those of the gold standard (complete diagnostic autopsy [CDA], which includes any available clinical information). Concordance between the MIA and the gold standard diagnostic categories was assessed by the kappa statistic, and the sensitivity, specificity, positive and negative predictive values and their 95% confidence intervals (95% CI) to identify the categories of diagnoses were estimated. The main limitation of the study is that both the MIA and the CDA include some degree of subjective interpretation in the attribution of cause of death.

A cause of death was identified in the CDA in 98% (56/57) of cases, with indirect obstetric conditions accounting for 32 (56%) deaths and direct obstetric complications for 24 (42%) deaths. Nonobstetric infectious diseases (22/32, 69%) and obstetric hemorrhage (13/24, 54%) were the most common causes of death among indirect and direct obstetric conditions, respectively. Thirty-six (63%) women were HIV positive, and HIV-related conditions accounted for 16 (28%) of all deaths. Cerebral malaria caused 4 (7%) deaths. The MIA identified a cause of death in 86% of women. The overall concordance of the MIA with the CDA was moderate (kappa = 0.48, 95% CI: 0.31–0.66). Both methods agreed in 68% of the diagnostic categories and the agreement was higher for indirect (91%) than for direct obstetric causes (38%). All HIV infections and cerebral malaria cases were identified in the MIA. The main limitation of the technique is its relatively low performance for identifying obstetric causes of death in the absence of clinical information.

**Conclusions:**

The MIA procedure could be a valuable tool to determine the causes of maternal death, especially for indirect obstetric conditions, most of which are infectious diseases.

The information provided by the MIA could help to prioritize interventions to reduce maternal mortality and to monitor progress towards achieving global health targets.

## Abstract 2

In an observational study, Clara Menéndez and colleagues assess the concordance of minimally invasive autopsy with complete diagnostic autopsy in cases of maternal death.

## Author summary

**Why was this study done?:**

- Since 1990, the maternal mortality ratio (MMR) has dropped by 43%, but despite this progress, hundreds of women still die every day in large parts of the world due to pregnancy or childbirth complications.
- A reliable knowledge of the causes of maternal death is a necessary condition to reduce this burden through adequate health planning.
- Current methods, such as verbal autopsies or the review of clinical data, have shown a high degree of misclassification in this specific group of deaths.
- We aimed to validate a minimally invasive autopsy (MIA) approach as a possible complement to verbal autopsies by comparing its performance against the complete diagnostic autopsy, the gold standard for cause of death investigation.

**What did the researchers do and find?:**

- We performed paired MIA and CDA in 57 maternal deaths that occurred at the Maputo Central Hospital, Mozambique, and assessed the concordance between both methods.
- Indirect obstetric diseases caused 56% of all deaths and direct obstetric complications accounted for 42% of deaths.
- Infectious diseases and obstetric hemorrhage were the most common causes of death among indirect and direct obstetric conditions, respectively.
- The overall concordance of the MIA with the CDA was moderate and both methods agreed in 68% of the diagnostic categories.
- The etiological microorganisms in infectious diseases causes of death were identified in 67% of the MIAs.

**What do these findings mean?:**

- The MIA procedure could be a valuable tool to determine the causes of maternal death, especially in indirect obstetric conditions, most of which are infectious diseases.
- This information may be helpful for decision-making on health planning and prioritization of interventions to reduce maternal mortality and for monitoring progress towards achieving global health targets.

## Introduction

As the Millennium Development Goals came to a close in 2015, the maternal mortality ratio (MMR) had dropped by 43% since 1990 [1]. Although this progress is certainly encouraging, it is lower than the 75% target initially planned, and hundreds of women still die every day due to complications of pregnancy or childbirth. In 2015, the number of estimated maternal deaths occurring worldwide was 303,000, most of which were preventable and disproportionately took place in low- and middle-income countries. The MMR in low-income countries in 2015 was 239 per 100,000 live births versus 12 per 100,000 live births in high-income countries, according to WHO [2]. In Mozambique, in 2015, the MMR was 489 per 100,000 live births, with an annual rate of reduction of 4.4% from 2005–2015 [3]. The aim of Sustainable Development Goal 3.1 is to reduce the global MMR to less than 70 per 100,000 live births by 2030 [4]. The accomplishment of this objective requires robust data sources to develop accurate estimates and, importantly, a thorough understanding of the causes of these deaths. Monitoring progress towards the 5th Millennium Development Goal, focusing on maternal health, revealed the lack of high-quality data in most countries, especially those with the highest MMR [1].

Recently, a minimally invasive autopsy (MIA) protocol has been adapted for cause of death determination in middle- and low-income settings [5]. This postmortem procedure, which consists of the sampling of key organs and fluids for histological and microbiological analysis, could add value to the currently used methods that rely on verbal autopsies and clinical records, which have been shown to have a high level of imprecision, especially for maternal and perinatal deaths [6,7].

The complete diagnostic autopsy (CDA) is the gold standard for cause of death determination but it is not free of limitations. The CDA is an invasive procedure, often not accepted by the relatives, and requires trained pathologists to perform it. The MIA, as opposed to the CDA, has been shown to be simple and more acceptable [8] and can be performed by less qualified personnel. The MIA has been recently validated in a series of in-hospital stillbirth, neonate, pediatric, and adult deaths in Mozambique [5]. These validation studies have shown that the MIA may reliably identify the cause of death with high concordance when compared with the gold standard CDA diagnosis, particularly for infectious diseases.

In this study, we analyzed the validity of the MIA to determine the cause of death in a series of in-hospital maternal deaths from Mozambique.

## Methods

### Study setting and design

This observational study received the approval of the Clinical Research Ethics Committee of the Hospital Clinic of Barcelona (Spain; approved, File 2013/8677) and the National Bioethics Committee of Mozambique (Mozambique; approved, Ref. 342/CNBS/13).

The study was conducted at the Departments of Gynecology and Obstetrics and Pathology of the Maputo Central Hospital, a 1,500-bed government-funded institution that serves as the referral center for other hospitals in southern Mozambique. Recruitment of maternal deaths was conducted from November 2013 to March 2015 as part of a comprehensive validation study of the validity of the MIA in different age groups [9].

We included in the study all deceased women fulfilling the following inclusion criteria: (1) death during pregnancy or within 42 days of termination of pregnancy, irrespective of its cause (maternal deaths, as defined by WHO) [10,11]; (2) a CDA requested by the clinician as part of the medical evaluation of the patient; and (3) oral informed consent to perform the autopsy given by the relatives. Accidental or incidental deaths were excluded.

The Strengthening the Reporting of Observational Studies in Epidemiology (STROBE) statement and the prospective analysis plan are included as supplementary information (S1 STROBE Checklist and S1 Text, respectively).

The overall study plan, indicating how postmortem procedures were performed, the laboratory investigations involved, and site and timing of each procedure, has been reported elsewhere [5].

### Autopsy procedures

Detailed MIA pathological and microbiological methods have been reported elsewhere [12,13]. The procedure, tailored for maternal deaths, included an initial disinfection of the surface of the body followed by the collection of blood and cerebrospinal fluid (CSF), aiming to collect about 20 mL. The procedure also included the puncture of solid organs (liver, lungs, and central nervous system [CNS]) for microbiological and pathological analysis using biopsy needles (14G–16G). In addition, the heart, spleen, and kidneys were targeted for pathology examination. In this particular group, biopsy samples of the uterus were attempted with a 14G biopsy needle, which were analyzed histologically and microbiologically. In addition, a sample of amniotic fluid, if available, was obtained.

Immediately after the MIA, the CDA procedure was conducted by a second pathologist not involved in the MIA and following a standardized protocol for maternal autopsies [5]. Histological and microbiological analyses were conducted in samples from the same viscera collected in the MIA and from any grossly identified lesions. The microbiological results of the blood and CSF were also included in the CDA evaluation.

### Histological and microbiological analyses

A team of 2 pathologists (J.O., P.Ca.) and 2 microbiologists (M.J.M., J.H.) reviewed and analyzed the samples from the MIA blindly to any clinical data except the information on the maternal status at death (pregnant, puerperal, previous abortion) that allowed classifying the case as a maternal death. All samples collected for histology were routinely stained with hematoxylin and eosin. Additional histochemical (e.g., Masson trichrome) and/or immunohistochemical stains and in situ hybridization techniques (e.g., Epstein–Barr virus-encoded small RNAs) were used, whenever needed, to reach a diagnosis. The microbiological methods have been reported in detail elsewhere [13]. In all patients, screening for context-epidemiologically common pathogens was conducted, including *Plasmodium falciparum* by real-time PCR detection of antibodies against human immunodeficiency virus (HIV)-1/2, hepatitis B virus surface antigen, and antibodies against hepatitis C virus. In all HIV-infected patients, the viral load was determined routinely. Additionally, *Toxoplasma gondii*, *Mycobacterium tuberculosis*, and *Cryptococcus* spp. in CSF and CNS samples and *Pneumocystis jirovecii*, *Cryptococcus* spp., and *M*. *tuberculosis* in the lungs were tested by real-time PCR. Any other microorganisms were only investigated depending on the pathological findings observed in the MIA-obtained tissues.

After a washout period of 3–6 months, the same team of experts analyzed the samples of the CDA following the same approach used for the analysis of the MIA samples.

### Determination of the cause of death

The methodology for cause of death determination has been detailed elsewhere [5]. Briefly, once all the analysis of the MIA samples had been completed, a panel composed of a pathologist, a microbiologist, an obstetrician, and an epidemiologist evaluated all the data of the MIA and assigned the MIA diagnosis, i.e., the disease or condition putatively leading to death. The participants in the MIA cause of death attribution panel were aware of the external macroscopic examination of the body (but not of the organs) and the histological and microbiological results obtained in the MIA sampling but were blind for the information on the clinical records, except for the pregnancy status and gestational age.

After a washout period (minimum time 3 months, range 3–6 months), the same panel evaluated the data from the CDA and assigned the CDA diagnosis of cause of death, which was considered the gold standard. As per routine practice in CDA procedures, investigators involved in the CDA diagnosis were aware of the clinical data, the macroscopic (external and internal) findings, and the histological and microbiological results. Using a combination of the strength of the evidence of the histological and the microbiological findings, a category was assigned to the certainty of the cause of death attribution of the MIA diagnosis and the CDA diagnosis (details published elsewhere) [5]. In this particular group, the obstetric history of severe intrapartum hemorrhage increased by 2 levels the strength of the pathological findings of the CDA.

All morbid conditions were codified following the International Classification of Diseases, 10th revision for maternal mortality (ICD-10 MM) [10,14]. Up to 4 diagnoses were established following the most probable sequence of events leading to death, starting from the immediate cause and going back to the earliest event, i.e., the underlying condition. Finally, other conditions or concomitant infections that may have existed prior to development of the underlying cause of death or developed during the chain of events leading to death and that, by their nature, contributed to the death, were considered as contributory conditions (e.g., multiple pregnancy) [10]. As indicated by WHO, all deaths were classified either as (1) direct obstetric deaths (i.e., those resulting from obstetric complications of the pregnancy, labor, or puerperium) or (2) indirect obstetric deaths (i.e., those resulting from previous existing diseases or diseases developed during pregnancy but not due to direct obstetric causes) [10].

Additionally, all deaths were aggregated in 8 categories according to the underlying causes of death as stated in the ICD-10 MM [10], which included the following: (1) pregnancies with abortive outcome; (2) hypertensive disorders in pregnancy, childbirth, and puerperium; (3) obstetric hemorrhage; (4) pregnancy-related infections; (5) other obstetric complications; (6) unanticipated complications of management; (7) nonobstetric complications; and (8) unexplained deaths. Nonobstetric complications include the following diseases: cardiovascular (including preexisting hypertension), endocrine, gastrointestinal tract, CNS, respiratory, genitourinary, autoimmune, skeletal, psychiatric diseases, neoplasm, and infections that are not a direct result of pregnancy. Categories 1 to 6 were considered direct obstetric deaths, and deaths classified as category 7 were considered indirect obstetric deaths.

### Statistical methods

The concordance between the MIA and the CDA diagnosis (gold standard) was established based on the concordance in the underlying cause of death categories, was assessed by the kappa statistic, and was interpreted as suggested by Landis and Koch [15]. The diagnostic performance of the MIA to identify the categories established by the gold standard diagnosis was evaluated in terms of sensitivity, specificity, positive and negative predictive values, and total percentage of cases correctly classified.

The level of coincidence between the MIA and the CDA diagnoses was analyzed by comparing the ICD-10 MM codes in order to identify the conditions that were concordant in the disease category but were not coincident in the main diagnosis (e.g., a case categorized as an indirect obstetric death in both methods but as pneumonia in the CDA and as sepsis in the MIA). As the ICD-10 system classifies diagnoses into nested classes of different hierarchical levels in which diseases or conditions are organized in chapters, blocks, and 3-character categories [14], a coincidence was classified as (i) perfect when the ICD-10 codes were identical in chapter, block, and 3-character categories; (ii) moderate when the codes were within the same chapter and block but there was a discrepancy in the 3-character category; or (iii) low when the codes were within the same chapter but not in the same block and 3-character categories. When the MIA and the CDA diagnoses were in different chapters, the coincidence was classified as “none.” The statistical analyses were performed using Stata version 14.1 (Stata, College Station, TX, USA). The analytical plan was determined once all the histological and microbiological results as well as the ICD10-MM codes were available.

## Results

Coupled MIA and CDA procedures were performed in 57 women. Median age was 27 years (range 15–39 years). Thirty-one women (54%) died in the puerperal period, 9 (16%) died after an abortion, and 16 (28%) were pregnant at the time of death. In all 16 cases, the fetus and the placenta were in situ. Nevertheless, no autopsy was performed on these in situ fetuses because it was not considered as relevant to determine the cause of death. The placenta was analyzed as part of the CDA in 8 of these cases but was not contributory to the final diagnosis. Forty-four lived in the Maputo urban area, whereas 13 were transferred from a rural district.

In 40 cases (70%), the interval between death and the beginning of postmortem procedures was ≤24 hours, whereas in 17/57 (30%), it ranged between 25 and 64 hours. The age of the patients, time from death to procedure, HIV status, MIA and CDA diagnoses, ICD-10 coding, level of certainty, and concordance between both diagnoses are shown for all patients in S1 Table.

Blood was obtained in all cases, but in 3 women, less than 10 mL were obtained (in 1 of these cases, the MIA was done 34 hours after death). In 56/57 cases (98%), 10 mL of CSF were obtained. In the MIA procedure, liver, CNS, and bone marrow samples were obtained in 100% of the cases, whereas lung was sampled in 93%, heart in 86%, spleen in 79%, and kidneys in 61% of the cases. The uterus was successfully obtained in 53/57 (93%) of the cases. Amniotic fluid was obtained in 7 (12%) cases. The placenta was available for analysis in 8/16 (50%) women that were pregnant at the time of death.

S1 Table summarizes the cause of death for each patient according to each of the 2 methods used.

### Gold standard (CDA) diagnosis of cause of death

A cause of death was identified in the CDA in 98% (56/57) of cases. The level of certainty of the CDA diagnosis was considered high or very high in 51/57 (89%) cases and moderate in 5 cases (9%).

Direct obstetric conditions accounted for 24 out of 57 deaths (42%). This included obstetric hemorrhages (13/24; 54%), complications of abortive pregnancies (6/24; 25%), pregnancy-related infections (4/24; 17%), and hypertensive disorders of the pregnancy (1/24; 4%) (Table 1, S1 Table). No deaths were classified in the categories “other obstetric complications” and “unanticipated complications of management”.

**Table 1:** Concordance of the cause of death categorization between the MIA and the CDA (gold standard).

MIA diagnosis	CDA diagnosis						
	Pregnancies with abortive outcome	Hypertensive disorders in pregnancy	Obstetric hemorrhage	Pregnancy-related infections	Nonobstetric complications	Non- conclusive	Total
**Pregnancies** **with abortive** **outcome**	**5**	0	0	0	1	0	**6 (11%)**
**Hypertensive** **disorders in** **pregnancy**	0	**1**	0	0	0	0	**1 (2%)**
**Obstetric** **hemorrhage**	0	0	**1**	0	0	0	**1 (2%)**
**Pregnancy-** **related** **infections**	0	0	1	**2**	0	0	**3 (5%)**
**Nonobstetric complications**	0	0	6	2	**29**	0	**37 (65%)**
**Non-** **conclusive**	1	0	5	0	2	**1**	**9 (16%)**
**Total**	**6 (11%)**	**1 (2%)**	**13 (23%)**	**4 (7%)**	**32 (56%)**	**1 (2%)**	**57 (100%)**

Kappa statistic: 0.48 (moderate agreement); 95% CI: 0.31–0.66.

**Abbreviations:** CDA, complete diagnostic autopsy; MIA, minimally invasive autopsy.

Indirect obstetric conditions accounted for 32/57 (56%) deaths. Among them, infectious diseases accounted for 22/32 deaths (69%), other diseases not related to pregnancy for 7/32 (22%), and malignant tumors for 3/32 (9%) of the deaths (Table 1, S1 Table). Five women died of tuberculosis (*M*. *tuberculosis)* and 4 of cryptococcal infection. Cerebral malaria with histological evidence of sequestration of parasitized erythrocytes in the capillaries of the CNS and *P*. *falciparum* detected by PCR was diagnosed in 4 women. Of these 4 patients, 3 (out of 44; 6.8%) came from the Maputo urban area, whereas 1 (out of 13; 7.7%) was transferred from a rural district.

HIV was detected in 36 women (63%). All HIV positive cases showed detectable viral load for HIV-1. HIV infection was identified in 13/24 (54%) women who died from direct obstetric causes and in 22/32 (69%) women who died from nonobstetric complications. HIV was also detected in the single case with nonconclusive diagnosis. Nearly 3 quarters (16/22; 73%) of the HIV-positive nonobstetric maternal deaths were considered to be AIDS related, which included all 5 tuberculosis cases, the 4 cryptococcal disseminated infections, 3 pneumonias, 1 streptococcal sepsis, 1 pyelonephritis, 1 meningoencephalitis, and 1 Burkitt lymphoma. The other 6 HIV-positive cases died from cerebral malaria (3 cases), liver failure due to massive liver necrosis (2 cases), and dilated cardiomyopathy (1 case). Hepatitis B virus infection was identified in 3 HIV-positive cases.

### MIA diagnosis and concordance in the categorization of disease between the MIA and the CDA gold standard diagnosis

A cause of death was identified in the MIA in 48 out of the 57 cases (84%). In 9 cases (16%), the MIA diagnosis was nonconclusive. The certainty of the MIA diagnosis was high or very high in 22/48 (46%) cases, moderate in 19/48 (40%), and low in 7/48 (14%).

Table 1 shows the diagnostic concordance between the MIA and the CDA (gold standard) for the categories of causes of death. The 2 procedures agreed in the diagnostic categories in 39/57 cases (68%) and the concordance was moderate according to the kappa statistics (kappa = 0.48, 95% CI: 0.31–0.66).

A perfect or almost perfect coincidence in the cause of death established by each method was observed in 36 of the 39 cases that were concordant in the diagnostic category. One case showed a low level of coincidence (diagnosed as chronic hypertensive disease in pregnancy in the CDA and as suggestive of cardiovascular disease in the MIA). The 2 remaining cases were noncoincident (a case of pneumonia and a case of liver necrosis according to the CDA, diagnosed as sepsis due to *Escherichia coli* and pneumonia, respectively, in the MIA).

Table 2 shows the sensitivity, specificity, and the positive and negative predictive values of the MIA diagnosis for the major diagnostic categories, as well as the percentage of false-positive and false-negative diagnoses, and the cases correctly classified (accuracy) in the MIA. The sensitivity of the MIA was very high for indirect causes of death (91%) and low (<10%) for obstetric hemorrhage. Overall, the accuracy of the MIA was at least 79% for all diagnostic categories.

**Table 2:** Sensitivity, specificity, positive and negative predictive value, and accuracy of the MIA for the different diagnostic categories in maternal deaths. Figures are percentages and 95% CI. S1 Table summarizes the cause of death for each patient according to each of the 2 methods used.

Causes of death in the CDA (gold standard)	Cases	Sensitivity (95% CI)	Specificity (95% CI)	Positive predictive value (95%CI)	Negative predictive value (95% CI)	Correctly classified (95% CI)
**Pregnancies with abortive outcome**	6	83 (36–100)	98 (90–100)	83 (36–100)	98 (90–100)	96 (88–100)
**Hypertensive disorders**	1	100 (2–100)	100 (94–100)	100 (2–100)	96 (94–100)	100 (94–100)
**Obstetric hemorrhage**	13	8 (0–36)	100 (92–100)	100 (2–100)	79 (66–88)	79 (66–88)
**Pregnancy-related infections**	4	50 (7–93)	98 (90–100)	67 (9–99)	96 (87–100)	95 (85–99)
**Nonobstetric complications**	32	91 (75–98)	68 (46–85)	78 (62–90)	85 (62–97)	81 (68–90)
**Nonconclusive**	1	100 (2–100)	86 (74–94)	11 (0–48)	100 (93–100)	86 (74–94)

**Abbreviations:** CDA, complete diagnostic autopsy; MIA, minimally invasive autopsy.

### Etiological agents in infectious diseases causes of death

Overall, infectious diseases accounted for 30/57 (57%) of maternal deaths. Infectious diseases included 8 obstetric infections (4 puerperal sepsis and 4 septic abortions) and 22 nonobstetric infections. An etiologic agent was identified in the CDA in 22 out of the 30 (73%) infectious diseases (6 obstetric and 16 nonobstetric). The same microorganism was identified in both the CDA and the MIA in 20 out of the 22 cases (91%). A microorganism was identified in the MIA in 13 additional cases and considered the MIA cause of death. In 5 cases, both the CDA and the MIA diagnosed an infectious disease, but whereas the CDA did not identify any microorganism, the MIA identified a causative agent. In 6 cases, the CDA (gold standard) cause of death was hemorrhagic shock and, consequently, the microorganism identified in the MIA was considered as not contributing to the death. Finally, in 2 cases, the CDA diagnosed an infectious disease, probably of bacterial origin, with no agent identifiable (a meningoencephalitis and a puerperal sepsis, respectively), whereas in the MIA the cause of death was considered to be a disseminated cytomegalovirus infection. In both cases, the cytomegalovirus was also identified in the CDA but it was not considered as the cause of death. Table 3 shows the etiological agents identified in the CDA and in the MIA.

**Table 3:** Etiological microorganisms identified in the CDA (gold standard) and in the MIA, and the number identified in both methods.

Etiological microorganisms	Microorganisms identified in the CDA (gold standard)	Microorganisms identified in the MIA and in the CDA	Microorganisms identified in the MIA but not in the CDA
**Direct obstetric infections**			
**Septic abortion**			
Enterobacteriaceae*	2	2	-
*Escherichia coli*	1	1	-
*Mycoplasma hominis*	1	1	-
*Aeromonas sobria*^†^	0	0	1
*Staphylococcus aureus*	0	0	1
**Puerperal sepsis**			
*S*. *pyogenes*	1	1	-
*Prevotella* spp.	1	0	-
*Aeromonas* spp.^†^	0	0	1
**Nonobstetric infections**			
**Disseminated infection**			
*Mycobacterium tuberculosis*	4	4	-
*Cryptococcus* spp.	3	3	-
*S*. *pneumoniae*	1	1	-
*Klebsiella pneumoniae*	1	1	2
*Cytomegalovirus*^†^	0	0	3
Enterobacteriaceae*^,^^††^	0	0	3
*E*. *coli*	0	0	1
*Toxoplasma gondii*	0	0	1
**Pulmonary infection**			
*S*. *aureus*	1	1	-
*M*. *tuberculosis*	1	0	-
**Central nervous system infection**			
*Plasmodium falciparum*	4	4	-
*Cryptococcus* spp.	1	1	-
**Total**	**22**	**20**	**13**

* Mixed infection of at least 2 of these enterobacteria: *Enterobacter cloacae*, *E*. *coli*, or *K*. *pneumoniae*

^†^ All the microorganisms identified in the MIA were also identified in the CDA but by definition were not considered as the cause of death.

^††^ One of the enterobacteria identified in the MIA was also identified in the CDA but was not considered the cause of death.

**Abbreviations:** CDA, complete diagnostic autopsy; MIA, minimally invasive autopsy.

## Discussion

This validation study shows, for the first time to our knowledge, that the MIA, a simplified postmortem procedure, can provide an acceptable correlation with the gold standard CDA diagnosis in maternal deaths. In this group, the agreement of the technique was 68% (kappa statistic: 0.48, 95% CI 0.31–0.66), a percentage of agreement similar to those observed in other age groups [16,17]. These findings suggest that this new method could provide reliable and relevant data regarding the causes of mortality associated with pregnancy and childbirth, particularly for indirect obstetric deaths, and thus contribute to reducing maternal mortality in the settings where this burden is highest.

This study was not designed to describe the causes of maternal death. However, our findings are similar to previous reports indicating that obstetric hemorrhage and indirect obstetric conditions are the leading causes of maternal mortality in low-income countries [18,19]. Interestingly, and unlike what it is usually reported [20,21], the proportion of indirect obstetric deaths (56%) observed in our study is larger than that of direct obstetric deaths (42%). These results are similar to a previous study carried out over 10 years ago in the same setting [19] and suggest that nonobstetric complications may be missed using the current methodologies and sources of data collection utilized for maternal cause of death estimation. Our study also confirmed the significant contribution of malaria and HIV to maternal mortality in endemic countries such as Mozambique [1,19,22,23]. In this series, malaria accounted for 7% (4/57) and HIV-related conditions for 28% (16/57) of all maternal deaths. Cerebral malaria could be detected in the MIA in all 4 cases [24], a finding of particular relevance, taking into account that the study was conducted in an urban setting where malaria transmission is known to be minimal. Interestingly, 3 out of the 4 women with cerebral malaria were of urban origin, although we cannot exclude a visit to a rural area with higher malaria transmission during pregnancy. This finding is very relevant for improving the knowledge on the impact of malaria on maternal mortality in endemic areas, which is usually underestimated [25]. As observed in previous reports [19,26,27], HIV and tuberculosis coinfection were found to be important causes of indirect obstetric deaths (9% of deaths). Tuberculosis was identified in the MIA in 4 out of 5 cases in which the infection was determined to be the cause of death in the CDA. This is of relevance given the low sensitivity of the clinical diagnosis of tuberculosis as a cause of maternal mortality [28]. Importantly, disseminated cryptococcal infection contributed significantly to death among HIV-positive women (7%, 4/57), and all the cases were also identified in the MIA. *Cryptococcus* spp. is the leading cause of adult meningitis in sub-Saharan Africa, where it is estimated to cause 15%–20% of all AIDS-related deaths [28–30]. Nevertheless, data in pregnant women are scarce, with only a few published studies having reported cryptococcal infection among pregnant women. This study highlights the importance of cryptococcosis as a cause of maternal death and thus the need to improve the diagnosis and management of this fungal infection during pregnancy [31–33].

Interestingly, the sensitivity of the MIA procedure was very high (91%) for indirect causes of death. In contrast, the sensitivity was much lower (36%) for direct obstetric causes. The sensitivity of the MIA procedure was particularly poor (less than 10%) for obstetric hemorrhage. Only 1 case was captured in the MIA because a retained placenta was identified in a woman during puerperium. Although only 1 death was attributed to eclampsia, the disease was identified in the MIA. This accuracy was probably influenced by our strict criteria to diagnose eclampsia, which required the presence of the typical pathological lesions in the liver and the absence of any other lethal lesion, in order to avoid overassignment of this condition as a cause of death [28]. This is relevant given the high number of false-positive clinical diagnoses of eclampsia that has been reported and the likely overestimation of this condition in clinical reports and verbal autopsies [34,35]. Indeed, it is essential to establish thoroughly the contribution of eclampsia to maternal mortality to guide maternal health programs that are based on preeclampsia prevention.

This study was designed to determine the validity of the MIA procedure by itself, without using any additional clinical data. The only exception to this purist approach was the knowledge of whether the death of the woman occurred while pregnant or within 42 days of termination of pregnancy (irrespective of the duration and the site of the pregnancy), as this information was essential to know when a case fulfilled the definition of WHO and, consequently, had to be included as a maternal death. Our findings indicate that the MIA without any clinical data has a relatively limited performance for direct obstetric causes of death. Some degree of obstetric information from the clinical record or the verbal autopsy could significantly improve the results of the MIA. Importantly, obstetric hemorrhage was missed in all but 1 case in the MIA; however, this condition is easily identified in the clinical records and is likely not to be difficult to retrieve from a verbal autopsy. On the contrary, most indirect obstetric deaths that are frequently misclassified in verbal autopsies were identified in the MIA. Future research should focus on the performance of the MIA with the inclusion of clinical data.

The extensive microbiological sampling and analysis proposed in our MIA protocol result in the adequate identification of the etiological agent in a high number of infectious diseases. Moreover, in 5 cases considered as infectious by the gold standard, the etiological agent was successfully identified in the MIA but not in the CDA. It is possible that the less invasive sampling procedure performed in the MIA might improve the performance of the microbiological analysis by reducing the probability of microbiological contamination [36,37]. On the other hand, the MIA may result, in the absence of any clinical information, in an overestimation of the microbiological results, as it may be the cases of the 5 women who died of obstetric hemorrhage that were considered as infectious deaths in the MIA.

A limitation of this study is the relatively low sample size, which has resulted in a poor representation of some causes of death. Nevertheless, the study was not designed to describe all the causes of maternal death and validate the method for each specific cause or category of disease but rather to assess the validity of the method for the group of maternal death as a whole. A second limitation of this study is that its diagnostic accuracy of the MIA could have been influenced by the dissemination of many diseases. More than half of the patients were HIV-infected adults with highly disseminated infections, and the performance of the procedure could be significantly reduced in focal lesions and in limited infections in immunocompetent hosts. Finally, both the MIA and the CDA include some degree of subjective interpretation in the attribution of cause of death.

In conclusion, establishing reliably the causes of maternal deaths is crucial for health planning and prioritization, which in turn are essential elements to reduce maternal mortality. The lack of quality data on these causes of death in high-burden countries has been recognized as a major limitation to achieving the 5th Millennium Developmental Goal [38], especially in sub-Saharan Africa, where the MMR only dropped by 26% since 1990 [1]. In these settings, a tool such as the MIA might provide quality information mainly for those conditions that are more difficult to identify through routinely used methods for cause of death determination, while improving clinical management and verbal autopsies with refined algorithms. This would lead to improving the most underachieving global health goal, which relates to maternal health.

## Supporting information

S1 STROBE Checklist Strengthening the Reporting of Observational Studies in Epidemiology (STROBE) checklist for the observational study developed in Mozambique to validate the minimally invasive autopsy for cause of death determination in maternal deaths. (DOCX)

S1 Table Gold standard and minimally invasive autopsy results of each case. The table includes the concordance between the 2 methods in terms of the disease category and the coincidence in terms of International Classification of Diseases, 10th revision for maternal mortality (ICD-10 MM) coding hierarchy.
(DOCX)

S1 Text Prospective analysis plan on pathological and microbiological procedures used during the study. (DOCX)

## Abbreviations

CDA: complete diagnostic autopsy
CI: confidence interval
CNS: central nervous system
CSF: cerebrospinal fluid
ICD-10 MM: International Classification of Diseases, 10th revision for maternal mortality
MIA: minimally invasive autopsy
MMR: maternal mortality ratio
