# Supplementary material for: Validity of a minimally invasive autopsy for cause of death determination in maternal deaths in Mozambique: An observational study
Source: PLoS Med. 2017 Nov 8;14(11):e1002431. doi: 10.1371/journal.pmed.1002431 (PMC5695595; doi:10.1371/journal.pmed.1002431)
Supplement: S1 Table — The table includes the concordance between the 2 methods in terms of the disease category and the coincidence in terms of International Classification of Diseases, 10th revision for maternal mortality (ICD-10 MM) coding hierarchy. (DOCX) [file pmed.1002431.s002.docx]

|  |  |  | | | | | **Causes of death** | | | | | | |  |  |  |
| --- | --- | --- | --- | --- | --- | --- | --- | --- | --- | --- | --- | --- | --- | --- | --- | --- |
|  |  | **General characteristics** | | | | | **Complete diagnostic autopsy (CDA, gold standard)** | | | **Minimally invasive autopsy (MIA)** | | | |  |  |  |
| **Case** | **Time from death to MIA (hours)** | **Age** | | **HIV status*** | | | **CDA diagnosis** | **ICD-10** | **Level of certainty** | **MIA diagnosis** | | **ICD-10** | **Level of certainty** | **Concordance in disease category** | **Coincidence in ICD-10 MM hierarchy** |  |
| **Pregnancies with abortive outcome** | | | | | | | |  |  |  | |  |  |  |  |  |
| 1 | 22 | 19 | Negative | | | | Septic abortion (*Escherichia coli*) | O08.0 | Very high (4+3) | Septic abortion (*Escherichia coli*) | | O08.0 | Moderate (2+2) | Yes | Perfect |  |
| 2 | 34 | 27 | Positive | | | | Septic abortion (*Mycoplasma hominis*) | O08.0 | Very high (4+4) | Septic abortion (*Mycoplasma hominis*) | | O08.0 | Moderate (1+4) | Yes | Perfect |  |
| 3 | 22 | 27 | Negative | | | | Hemorrhagic shock after incomplete abortion ^†^ | O08.1 | High (4+1) | Septic abortion (*Aeromonas* spp.) | | O08.0 | Moderate (1+4) | Yes | Almost perfect |  |
| 4 | 25 | 27 | Positive | | | | Septic abortion (*Enterobacteriaceae*) | O08.0 | Very high (4+4) | Septic abortion (*Enterobacteriaceae*) | | O08.0 | Moderate (1+3) | Yes | Perfect |  |
| 5 | 39 | 35 | Positive | | | | Septic abortion (*Enterobacteriaceae*) | O08.0 | Moderate (1+3) | Septic abortion (*Enterobacteriaceae*) | | O08.0 | Moderate (1+4) | Yes | Perfect |  |
| 6 | 5 | 31 | Negative | | | | Hemorrhagic shock secondary to abdominal pregnancy | O08.3 | High (4+0) | Non conclusive | | R99 | No diagnosis (0+0) | No | None |  |
| **Hypertensive disorders in pregnancy, childbirth, and puerperium** | | | | | | | | | | | | | | | | |
| 7 | 19 | 37 | | Positive | | | Cerebral hemorrhage secondary to eclampsia | O15.9 | High (4+0) | Liver changes of eclampsia | | O15.9 | Low (2+0) | Yes | Perfect |  |
| **Obstetric hemorrhage** | | | | | | | | | | | | | | | | |
| 8 | 16 | 22 | | Negative | | Premature separation of placenta with coagulation defect ^†^ | | O45.0 | High (4+1) | Sepsis *(Enterobacteriaceae)* | | A41.5 | Low (2+1) | No | None |  |
| 9 | 25 | 32 | | Positive | | Hemorrhagic shock secondary to retained placenta total | | O72.0 | High (4+0) | Retained placenta | | O72.0 | Moderate (3+0) | Yes | Perfect |  |
| 10 | 24 | 39 | | Positive | | Hemorrhagic shock secondary to uterine rupture ^‡^ | | O71.1 | High (4+1) | Suggestive of complications of hepatic failure due to cirrhosis ^§^ | | K72.9 | Moderate (1+4) | No | None |  |
| 11 | 18 | 15 | | Negative | | Hemorrhagic shock secondary to uterine atony | | O72.1 | High (4+0) | Non conclusive | | R99 | No diagnosis (0+0) | No | None |  |
| 12 | 4 | 18 | | Negative | | Hemorrhagic shock secondary to placental abruption ^†^ | | O45.9 | High (4+0) | Puerperal sepsis (*Aeromonas* spp.) | | O85 | Very high (4+4) | No | Low |  |
| 13 | 11 | 23 | | Negative | | Hemorrhage due to labor complication | | O67.9 | High (4+0) | Suggestive of cardiovascular disease | | O99.4 | Low (2+1) | No | Low |  |
| 14 | 29 | 24 | | Positive | | Hemorrhagic shock during and post cesarean section sec to placental abruption | | O45.9 | High (4+0) | Non conclusive | | R99 | No diagnosis (0+0) | No | None |  |
| 15 | 14 | 35 | | Positive | | Hemorrhagic shock post-partum secondary to vaginal laceration ^‖^ | | O71.4 | High (4+1) | Disseminated CMV | | B20.2 | Moderate (1+4) | No | None |  |
| 16 | 12 | 28 | | Positive | | Hemorrhagic shock secondary to uterine atony | | O72.1 | High (4+0) | Non conclusive | | R99 | No diagnosis (0+0) | No | None |  |
| 17 | 18 | 27 | | Negative | | Hemorrhagic shock secondary to atony uterine post placental abruption | | O72.1 | High (4+0) | Non conclusive | | R99 | No diagnosis (0+0) | No | None |  |
| 18 | 43 | 25 | | Positive | | Hemorrhagic shock post-hysterectomy secondary to placenta accreta^¶^ | | O72.0 | High (4+0) | Sepsis (*Enterobacteriaceae*) | | B20.1 | High (2+3) | No | None |  |
| 19 | 28 | 16 | | Negative | | Hemorrhagic shock secondary to retained placenta^¶^ | | O72.0 | High (4+0) | Sepsis (*Klebsiella pneumoniae*) | | A41.5 | Moderate (1+4) | No | None |  |
| 20 | 8 | 32 | | Negative | | Hemorrhagic shock secondary to atony uterine post-cesarean section secondary to multiple pregnancy | | O72.1 | High (4+0) | Non conclusive | | R99 | No diagnosis (0+0) | No | None |  |
| **Pregnancy-related infections** | | | | | | | |  |  |  | |  |  |  |  |  |
| 21 | 11 | 22 | | Negative | | Puerperal sepsis (*Prevotella* spp.) | | O85 | High (4+2) | Puerperal sepsis (no agent) | | O85 | Moderate (3+1) | Yes | Perfect |  |
| 22 | 34 | 27 | | Positive | | Puerperal sepsis (*Streptococcus pyogenes*) | | O85 | Moderate (1+3) | Puerperal sepsis (*Streptococcus pyogenes*) | | O85 | Moderate (1+3) | Yes | Perfect |  |
| 23 | 27 | 27 | | Positive | | Puerperal sepsis (no agent) ^¶^ | | O85 | High (4+0) | Sepsis (*Enterobacteriaceae*) | | B20.1 | Moderate (1+3) | No | None |  |
| 24 | 8 | 27 | | Positive | | Puerperal sepsis (no agent) ^‖^ | | O85 | High (4+1) | Disseminated CMV | | B20.2 | Moderate (1+4) | No | None |  |
| **Non-obstetric complications** | | | | | | | | | | | | | | | | |
| 25 | 9 | 28 | | Positive | Cerebral malaria | | | B50.0 | Very high (4+4) | Cerebral malaria | B50.0 | | Very high (3+4) | Yes | Perfect |  |
| 26 | 24 | 20 | | Negative | Cerebral malaria | | | B50.0 | Very high (4+4) | Cerebral malaria | B50.0 | | Very high (4+4) | Yes | perfect |  |
| 27 | 25 | 28 | | Positive | Cerebral malaria | | | B50.0 | Very high (4+4) | Cerebral malaria | B50.0 | | Very high (3+4) | Yes | Perfect |  |
| 28 | 9 | 23 | | Positive | Cerebral malaria | | | B50.0 | Very high (4+4) | Cerebral malaria | B50.0 | | Very high (4+4) | Yes | Perfect |  |
| 29 | 4 | 37 | | Positive | Miliary tuberculosis | | | B20.0 | Very high (4+4) | Miliary tuberculosis | B20.0 | | High (4+1) | Yes | Perfect |  |
| 30 | 11 | 28 | | Positive | Miliary tuberculosis | | | B20.0 | Very high (4+4) | Miliary tuberculosis | B20.0 | | Very high (4+4) | Yes | Perfect |  |
| 31 | 43 | 39 | | Positive | Miliary tuberculosis | | | B20.0 | Very high (4+4) | Miliary tuberculosis | B20.0 | | Very high (4+4) | Yes | Perfect |  |
| 32 | 14 | 32 | | Positive | Miliary tuberculosis | | | B20.0 | Very high (4+4) | Miliary tuberculosis | B20.0 | | Very high (4+4) | Yes | Perfect |  |
| 33 | 18 | 25 | | Positive | Pulmonary tuberculosis | | | B20.0 | Very high (4+4) | Pneumonia (no agent) | B20.9 | | High (4+1) | Yes | Almost perfect |  |
| 34 | 8 | 30 | | Positive | Meningoencephalitis (*Cryptococcus* spp.*)* | | | B20.5 | Very high (4+4) | Meningoencephalitis *(Cryptococcus* spp.*)* | B20.5 | | Very high (4+4) | Yes | Perfect |  |
| 35 | 24 | 21 | | Positive | Disseminated infection (*Cryptococcus* spp.) | | | B20.5 | Very high (4+4) | Disseminated infection (*Cryptococcus* spp.) | B20.5 | | Very high (4+4) | Yes | Perfect |  |
| 36 | 20 | 34 | | Positive | Disseminated infection (*Cryptococcus* spp.) | | | B20.5 | High (4+0) | Disseminated infection (*Cryptococcus* spp.) | B20.5 | | Very high (4+4) | Yes | Perfect |  |
| 37 | 8 | 31 | | Positive | Disseminated infection (*Cryptococcus* spp.) | | | B20.5 | Very high (4+4) | Disseminated infection (*Cryptococcus* spp.) | B20.5 | | Very high (4+4) | Yes | Perfect |  |
| 38 | 20 | 34 | | Positive | Sepsis (*Streptococcus pneumoniae*) | | | B20.1 | Very high (4+3) | Pneumonia (*Streptococcus pneumoniae*) | B20.1 | | Very high (4+3) | Yes | Perfect |  |
| 39 | 25 | 16 | | Negative | Sepsis (*Klebsiella pneumoniae*) | | | A41.5 | Very high (4+3) | Sepsis (*Klebsiella pneumoniae*) | A41.5 | | High (3+3) | Yes | Perfect |  |
| 40 | 19 | 19 | | Negative | Sepsis (no agent) ^¶^ | | | A41.9 | High (4+1) | Sepsis (*Klebsiella pneumoniae*) | A41.5 | | Moderate (1+3) | Yes | Almost perfect |  |
| 41 | 8 | 35 | | Negative | Pneumonia. Pulmonary granulomatous disease (no agent)^¶^ | | | J18.9 | High (4+0) | Sepsis (*Escherichia coli*) | A41.9 | | Moderate (1+3) | Yes | None |  |
| 42 | 50 | 28 | | Positive | Pneumonia (no agent) | | | B20.9 | High (4+1) | Pneumonia (no agent) | B20.9 | | High (4+1) | Yes | Perfect |  |
| 43 | 27 | 34 | | Positive | Pneumonia (*Staphylococcus aureus*) | | | B20.1 | High (3+4) | Pneumonia (*Staphylococcus aureus*) | B20.1 | | Very high (3+4) | Yes | Perfect |  |
| 44 | 7 | 30 | | Positive | Pneumonia (no agent)^¶^ | | | B20.9 | Moderate (3+0) | Septic abortion (*Staphylococcus aureus*) | O08.0 | | Low (1+2) | No | None |  |
| 45 | 18 | 19 | | Positive | Meningoencephalitis (no agent) ^‖^ | | | B20.1 | Moderate (3+1) | Disseminated CMV | B20.2 | | Moderate (1+4) | Yes | Almost perfect^**^ |  |
| 46 | 8 | 25 | | Positive | Pyelonephritis (no agent) | | | B20.1 | High (4+0) | Toxoplasmosis | B20.8 | | Moderate (2+2) | Yes | Almost perfect^**^ |  |
| 47 | 17 | 28 | | Positive | Massive liver necrosis | | | K72.9 | Moderate (3+1) | Massive liver necrosis | K72.9 | | Moderate (3+1) | Yes | Perfect |  |
| 48 | 24 | 27 | | Positive | Liver necrosis | | | K72.9 | High (4+1) | Pneumonia (no agent) | B20.1 | | Very high (4+1) | Yes | None |  |
| 49 | 21 | 35 | | Positive | Dilated myocardiopathy complicating pregnancy | | | O99.4 | High (4+0) | Suggestive of cardiovascular disease | O99.4 | | Low (2+0) | Yes | Perfect |  |
| 50 | 12 | 24 | | Negative | Rheumatic valvular heart disease complicating pregnancy | | | O99.4 | High (4+0) | Suggestive of cardiovascular disease | O99.4 | | Low (2+0) | Yes | Perfect |  |
| 51 | 21 | 33 | | Negative | Chronic hypertensive disease with severe cerebral edema in puerperium | | | O10.9 | Moderate (3+0) | Non conclusive | R99 | | No diagnosis (0+0) | No | None |  |
| 52 | 36 | 22 | | Negative | Chronic hypertensive disease in pregnancy (myocardiopathy hypertensive) | | | O10.9 | Moderate (3+0) | Suggestive of cardiovascular disease | O99.4 | | Low (2+1) | Yes | Low |  |
| 53 | 20 | 16 | | Negative | Liver necrosis secondary acute HBV hepatitis ^§^ | | | K72.9 | Very high (4+4) | Liver necrosis secondary to HBV hepatitis ^§^ | K72.9 | | Very high (3+4) | Yes | Perfect |  |
| 54 | 24 | 30 | | Positive | Burkitt's lymphoma ^††^ | | | B21.1 | High (4+4) | Burkitt's lymphoma ^††^ | B21.1 | | High (4+4) | Yes | Perfect |  |
| 55 | 26 | 32 | | Negative | Adenocarcinoma of the lung | | | C34.9 | High (4+0) | Non conclusive | R99 | | No diagnosis (0+0) | No | None |  |
| 56 | 23 | 21 | | Negative | Hepatocellular carcinoma | | | C22.9 | Very high (4+4) | Hepatocellular carcinoma | C22.9 | | Very high (4+4) | Yes | Perfect |  |
| **Unexplained deaths** | | | | | | | | | | | | | | | | |
| 57 | 64 | 36 | | Positive | | Non conclusive | | R99 | No diagnosis (0+0) | Non conclusive | | R99 | No diagnosis (0+0) | Yes | Perfect^‡‡^ |  |

*All HIV positive cases showed detectable viral load for HIV-1; †*Enterobacteriaceae* and *Aeromonas spp.* Identified in the CDA, but not considered the cause of death; ‡cirrhosis secondary to hepatitis B virus also identified in the liver biopsy; §Hepatitis B virus identified by molecular methods; ‖Cytomegalovirus identified in the CDA, but not considered the cause of death; ¶Microorganisms identified in the MIA were not identified in the CDA; **The coincidence is almost perfect since both pathologies are codified in the group of Human immunodeficiency virus [HIV] disease resulting in infectious and parasitic diseases ††Epstein Barr virus identified by in situ hybridization; ‡‡In both autopsies, all the samples showed severe autolysis in the histological exam.
